# Nomogram including indirect bilirubin for the prediction of post-stroke depression at 3 months after mild acute ischemic stroke onset

**DOI:** 10.3389/fneur.2023.1093146

**Published:** 2023-02-08

**Authors:** Yanyan Wang, Wenzhe Sun, Jinfeng Miao, Zhou Zhu, Wenwen Liang, Xiuli Qiu, Chensheng Pan, Guo Li, Yan Lan, Xin Zhao, Yi Xu

**Affiliations:** ^1^Department of Neurology, Tongji Hospital, Tongji Medical College, Huazhong University of Science and Technology, Wuhan, Hubei, China; ^2^Department of Plastic Surgery, Tongji Hospital, Tongji Medical College, Huazhong University of Science and Technology, Wuhan, Hubei, China

**Keywords:** indirect bilirubin, post-stroke depression, mild acute ischemic-stroke, nomogram, oxidative stress

## Abstract

**Background:**

Post-stroke depression (PSD) has been proven to be associated with stroke severity. Thus, we hypothesized that the prevalence of PSD would be lower in patients with mild stroke. We aim to explore predictors of depression at 3 months after mild acute ischemic stroke (MAIS) onset and to develop a practical and convenient prediction model for the early identification of patients at high risk.

**Methods:**

A total of 519 patients with MAIS were consecutively recruited from three hospitals in Wuhan city, Hubei province. MAIS was defined as a National Institute of Health Stroke Scale (NIHSS) score of ≤5 at admission. Meeting the DSM-V diagnostic criteria and a 17-item Hamilton Rating Scale for Depression (HAMD-17) score of >7 at their 3-month follow-up were considered the primary outcomes. A multivariable logistic regression model was used to determine the factors adjusted for potential confounders, and all independent predictors were brought into the construction of a nomogram to predict PSD.

**Results:**

The prevalence of PSD is up to 32% at 3 months after MAIS onset. After adjusting for potential confounders, indirect bilirubin (*p* = 0.029), physical activity (*p* = 0.001), smoking (*p* = 0.025), hospitalization days (*p* = 0.014), neuroticism (*p* < 0.001), and MMSE (*p* < 0.001) remained independently and significantly related with PSD. The concordance index (C-index) of the nomogram jointly constructed by the aforementioned six factors was 0.723 (95% CI: 0.678–0.768).

**Conclusion:**

The prevalence of PSD seems equally high even if the ischemic stroke is mild, which calls for great concern from clinicians. In addition, our study found that a higher level of indirect bilirubin can lower the risk of PSD. This finding may provide a potential new approach to PSD treatment. Furthermore, the nomogram including bilirubin is convenient and practical to predict PSD after MAIS onset.

## Introduction

Post-stroke depression (PSD), as one of the most frequent psychiatric complications of cerebrovascular lesions, is closely linked to decreased functional status and higher mortality rates, which not only reduces the quality of life of patients but also places a heavy burden on caregivers (1–3). A new review reported that PSD prevalence was extremely high within 3 months after the acute event in the total stroke population (4), and our previously published literature showed that the overall prevalence of PSD at 3 months after stroke was up to 39.7% (5). Furthermore, a host of studies presented that there was a strong association between stroke severity and PSD (1, 2, 6).

In clinical practice, the National Institute of Health Stroke Scale (NIHSS) is widely used to evaluate the severity of stroke (7), and mild stroke was defined as an NIHSS score of ≤5 (8, 9). An interesting study showed that despite a low NIHSS, patients with mild stroke who have recovered well continue to experience psychological consequences such as PSD and fatigue (10). The prevalence of depression after a minor ischemic stroke has been reported to be 26% 1 year after the stroke onset (11). Without timely identification and proper treatment, patients with long-term mood disorders after mild stroke could have a worse quality of life and may find it difficult to return to work and their social activities (12).

Currently, the literature on predicting PSD after mild stroke is still relatively scarce, the lack of data in this field provided the impetus for the study reported herein. To this end, we examine the prevalence and explore independent predictors of depression at 3 months after mild acute ischemic stroke (MAIS) onset. Moreover, we are committed to establishing a practical predictive nomogram of PSD to guide clinical decision-making.

## Methods

### Study design and subjects

As a multicenter prospective cohort study, our research was conducted at Tongji Hospital, Wuhan First Hospital, and Wuhan Central Hospital between August 2018 and June 2019. This study was approved by the ethics committee of Tongji Medical College, Huazhong University of Science and Technology (approval no. of ethics committee: TJ-IRB20171108). According to the Declaration of Helsinki, all subjects signed an informed consent form. Patients with MAIS were consecutively recruited in this study (*n* = 519).

### Inclusion and exclusion criteria

The inclusion criteria were as follows: (1) must be over 18 years of age and sign informed consent; (2) ischemic stroke was confirmed by clinical symptoms and computed tomography (CT) or magnetic resonance imaging (MRI) scans; (3) the onset time was <1 week; and (4) NIHSS score at admission was ≤5.

Exclusion criteria were as follows: (1) transient ischemic attack (TIA) and subarachnoid hemorrhage (SAH); (2) depression before the ischemic stroke, a history of antidepressants, and other mental illnesses such as schizophrenia; (3) history of severe hepatobiliary diseases, kidney failure, and hemolytic disease; (4) dementia and cognitive dysfunction [Mini-Mental State Examination (MMSE) score <17 points]; (5) Parkinson's disease, epilepsy, and other concomitant neuropsychiatric diseases; (6) non-vascular causes resulted in brain disorder, such as brain trauma; (7) deafness, blindness, and aphasia; and (8) failure to complete follow-up.

### Data collection

Patient's sociodemographic characteristics and previous medical history, including age, sex, height, weight, physical activity, smoking, drinking, hypertension, hyperlipemia, diabetes mellitus (DM), transient ischemic attack (TIA), coronary heart disease (CHD), and hospitalization days, were obtained from a comprehensive questionnaire.

Venous blood was drawn from patients the next morning after admission. We measured fasting c-peptide (FCP), total cholesterol (TC), triglyceride (TG), total bilirubin (TB), direct bilirubin (DB), serum indirect bilirubin (IB), cortisol, adrenocorticotropic hormone (ACTH), interleukin (IL), c-reactive protein (CRP), brain-derived neurotrophic factor (BDNF), tumor necrosis factor-α (TNF-α), and interferon (IFN-γ).

### Valid scales

The MMSE, the NIHSS, the Modified Rankin Scale (mRS), the Barthel index (BI), the Neuroticism Scale (the subscale of the Eysenck Personality Questionnaire), and the Conner-Davidson Resilience Scale (CD-RISC) were evaluated after admission. All of these scales have been shown to have good reliability and validity. The MMSE scale, which is widely used at home and abroad, is the preferred scale for screening cognitive dysfunction (13). The NIHSS is a reliable, valid, and responsive tool for measuring stroke severity; it is useful both in clinical practice and research (14). The mRS, a simple and convenient 7-point scale ranging from 0 (no symptoms at all) to 6 (death), is widely used to assess the severity of disability in patients with stroke (15, 16). The BI is often used to evaluate the participants' activities of daily living performance, which has been previously proven to have good reliability and validity and is widely used in the Chinese context (17). The Eysenck Personality Questionnaire, which consists of three dimensions of extraversion, psychoticism, and neuroticism, is widely used to assess a person's personality traits (18). The CD-RISC has been tested in the general population, as well as in clinical samples, and demonstrates sound psychometric properties, with good internal consistency and test–retest reliability (19).

The 17-item Hamilton Rating Scale for Depression (HAMD-17) has been proven to have good reliability and validity in the Chinese population (20), which therefore was used to measure the severity of depressive symptoms at 3, 6, and 12 months after MAIS onset (this article only presents the data analysis results of 3 months). A participant who met DSM-V diagnostic criteria (depression caused by other medical conditions) along with a HAMD-17 score of >7 was diagnosed with PSD.

### Imaging examination

The CT examination instrument was a GE64-slice spiral CT, the tube voltage was 120 KV, the tube current was 120 mA, the slice thickness was 10 mm, and the pitch was 10 mm.

MRI was performed with a GE 3.0T MR scanner (Discovery MR 750 System, GE Healthcare, Milwaukee, WI, USA) with an eight-channel head coil. Diffusion-weighted imaging (DWI) was mainly used to detect acute new cerebral infarction. DWI scanning parameters were as follows: repetition time was 3,000 ms, echo time was 65 ms, layer thickness was 5 mm, layer distance was 1.5 mm, an intra-layer resolution was 256 × 256, and the field of view was 240 × 240 mm^2^.

### Statistical analysis

The baseline characteristics of the patients were compared between the PSD group and the NPSD group. Medians with interquartile ranges or mean ± standard deviation were used for continuous variables. Frequencies with percentages were used for categorical variables. If the continuous variable conforms to the normal distribution, the *t*-test is adopted to test the statistical difference; otherwise, the Mann–Whitney *U*-test is adopted. As for categorical variables, χ^2^ tests were used to compare group differences. All tests were two-sided, and a *P*-value of 0.05 was defined to indicate statistical significance. Subsequently, variables with *p* < 0.05 were entered into a multivariable logistic regression model in a backward stepwise method (21). The above-mentioned analysis process was realized by Statistical Program for Social Sciences (SPSS) statistical software (version 25, Chicago, IL, USA). According to the results of multivariable logistic regression, a nomogram for the prediction of PSD at 3 months after ischemic stroke onset was established using the R package “rms” in R version 4.0.3 (http://www.r-project.org/).

## Results

### Baseline characteristics

In total, 519 patients with MAIS were consecutively recruited in this study. Of all these potential subjects, 166 (32%) developed PSD at 3 months after ischemic stroke onset. Table 1 shows a comparison of baseline information between the non-PSD and the PSD groups. There was no significant difference in age and sex between the two groups. But the PSD group had a lower proportion of smoking history (*p* = 0.014), a lower proportion of physical activity (*p* = 0.001), longer hospitalization days (*p* = 0.002), a higher NIHSS score (*p* < 0.001), a higher mRS score (*p* < 0.001), a lower MMSE score (*p* < 0.001), a lower BI score (*p* = 0.004), a lower CD-RISC score (*p* = 0.009), and a higher neuroticism score (*p* < 0.001). As for serum biochemicals, the levels of IB, FCP, and ACTH were found to be significantly different between the two groups. The PSD group showed a lower level of IB (*p* = 0.013), a higher level of FCP (*p* = 0.013), and a higher level of ACTH (*p* = 0.047).

**Table 1:** Demographic and clinical characteristics of patients without and with PSD at 3 months.

**Parameter**	**NPSD (*N* = 353) 68%**	**PSD (*N* = 166) 32%**	***P***
**Demographic information**			
Age, *X* ± SD	59.1 ± 11.5	58.1 ± 10.6	0.340
Female, *n* (%)	65 (18.4)	41 (24.7)	0.098
BMI, *X* ± SD	24.4 ± 3.0	24.5 ± 3.3	0.555
Hospitalization days, median (IQR)	9 (7, 12)	10 (8, 13)	0.002^*^
Physical activity, *n* (%)	156 (44.2)	47 (28.3)	0.001^*^
Smoking, *n* (%)	231 (65.4)	90 (54.2)	0.014^*^
Drinking, *n* (%)	201 (56.9)	88 (53.0)	0.401
Hypertension, *n* (%)	207 (58.6)	95 (57.2)	0.761
Diabetes mellitus, *n* (%)	101 (28.6)	49 (29.5)	0.832
Hyperlipemia, *n* (%)	96 (27.2)	45 (27.1)	0.983
TIA, *n* (%)	19 (5.3)	17 (10.0)	0.042^*^
CHD, *n* (%)	30 (8.5)	20 (12.0)	0.201
**Serum biochemicals**			
TC, median (IQR)	4.1 (3.4, 4.8)	4.0 (3.4, 4.8)	0.572
TG, median (IQR)	1.5 (1.0, 2.1)	1.4 (1.0, 2.1)	0.999
Total bilirubin, median (IQR)	12.9 (9.1, 15.2)	12.5 (8.8, 14.2)	0.071
Direct bilirubin, median (IQR)	4.1 (2.8, 4.9)	4.3 (2.9, 5.1)	0.248
Indirect bilirubin, median (IQR)	8.6 (5.9, 10.6)	7.7 (5.3, 9.2)	0.013^*^
FCP, median (IQR)	1.9 (1.0, 2.5)	2.0 (1.4, 2.6)	0.013^*^
ACTH, median (IQR)	27.5 (14.5, 44.5)	31.6 (18.9, 43.5)	0.047^*^
CRP, median (IQR)	1.8 (0.8, 4.9)	1.6 (0.8, 3.3)	0.583
Cortical, median (IQR)	12.5 (10.3, 15.2)	12.7 (10.0, 16.2)	0.442
IL.1β, median (IQR)	63.9 (25.6, 169.2)	63.1 (30.0, 164.6)	0.740
IL.6, median (IQR)	6.0 (3.23, 7.8)	6.0 (2.3, 12.4)	0.215
IL.10, median (IQR)	8.8 (2.6, 21.8)	8.9 (3.1, 21.9)	0.962
IL.18, median (IQR)	1,993.4 (945.4, 4436.2)	1,988.2 (833.9, 4977.3)	0.957
BDNF, median (IQR)	3.8 (2.2, 8.8)	3.6 (2.0, 7.2)	0.264
TNF.α, median (IQR)	40.4 (23.2, 61.2)	37.9 (19.7, 54.9)	0.128
IFN. γ, median (IQR)	4.2 (1.8, 8.4)	4.5 (2.0, 8.6)	0.368
**Clinical characteristics**			
NIHSS, *X* ± SD	2.0 ± 1.5	2.5 ± 1.4	<0.001^*^
BI, median (IQR)	100 (90, 100)	95 (79, 100)	0.004^*^
mRS, median (IQR)	1 (1, 2)	2 (1, 3)	<0.001^*^
MMSE, median (IQR)	27 (25, 29)	26 (22, 28)	<0.001^*^
CD-RISC, median (IQR)	65 (55, 79)	61 (51, 73)	0.009^*^
Neuroticism, median (IQR)	7 (4, 11)	10 (6, 14)	<0.001^*^

BMI, body mass index; TIA, transient ischemic attack; CHD, coronary heart disease; TC, total cholesterol; TG, triglyceride; FCP, fasting c-peptide; ACTH, adrenocorticotropic hormone; CRP, c-reactive protein; IL, interleukin; BDNF, brain-derived neurotrophic factor; TNF, tumor necrosis factor; IFN, interferon; NIHSS, national institutes of health stroke scale; BI, barthel index; mRS, modified rankin scale; MMSE, mini-mental state examination; CD-RISC, conner-davidson resilience scale; neuroticism scale, subscale of eysenck personality questionnaire.

^*^Statistically significant at p < 0.05 level, two-sided.

### Independent predictors of PSD

The multivariable logistic regression model shows that IB (OR = 0.945, 95% CI: 0.899–0.994, *p* = 0.029), physical activity (OR = 0.498, 95% CI: 0.327–0.756, *p* = 0.001), smoking (OR = 0.633, 95% CI: 0.425–0.945, *p* = 0.025), hospitalization days (OR = 1.050, 95% CI: 1.010–1.092, *p* = 0.014), neuroticism (OR = 1.083, 95% CI: 1.040–1.127, *p* < 0.001), and MMSE (OR = 0.906, 95% CI: 0.857–0.957, *p* < 0.001) were significantly and independently related with PSD at 3 months after the onset of ischemic stroke (Table 2).

**Table 2:** Multivariable logistic regression model for PSD at 3 months.

**Parameter**	***B***	**SE**	***P***	**OR (95% CI)**
Hospitalization days	0.049	0.020	0.014^*^	1.050 (1.010–1.092)
Physical activity	−0.698	0.213	0.001^*^	0.498 (0.327–0.756)
Smoking	−0.457	0.204	0.025^*^	0.633 (0.425–0.945)
Indirect bilirubin	−0.056	0.026	0.029^*^	0.945 (0.899–0.994)
Neuroticism	0.080	0.021	0.000^*^	1.083 (1.040–1.127)
MMSE	−0.099	0.028	0.000^*^	0.906 (0.857–0.957)

MMSE, mini-mental state examination; SE, standard error; OR, odds ratio; CI, confidence interval.

^*^Statistically significant at p < 0.05 level, two-sided.

### Nomogram for the prediction of PSD

**Figure 1:**
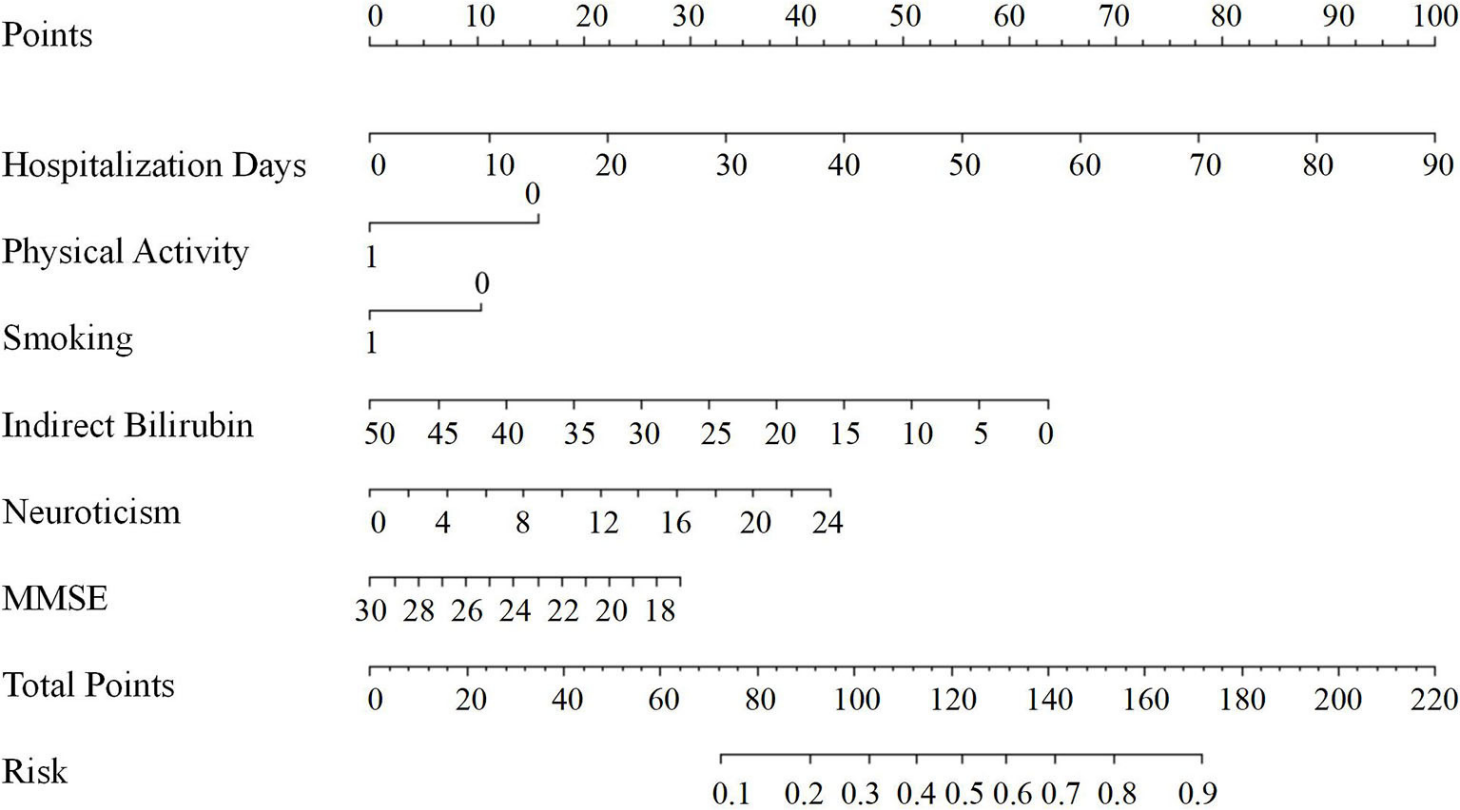
Nomogram for predicting PSD at 3 months after MAIS onset. The final score (i.e., total points) is calculated as the sum of the individual score of each of the six variables included in the nomogram. MMSE, the mini-mental state examination; Risk, the risk of PSD at 3 months after MAIS.

**Figure 2:**
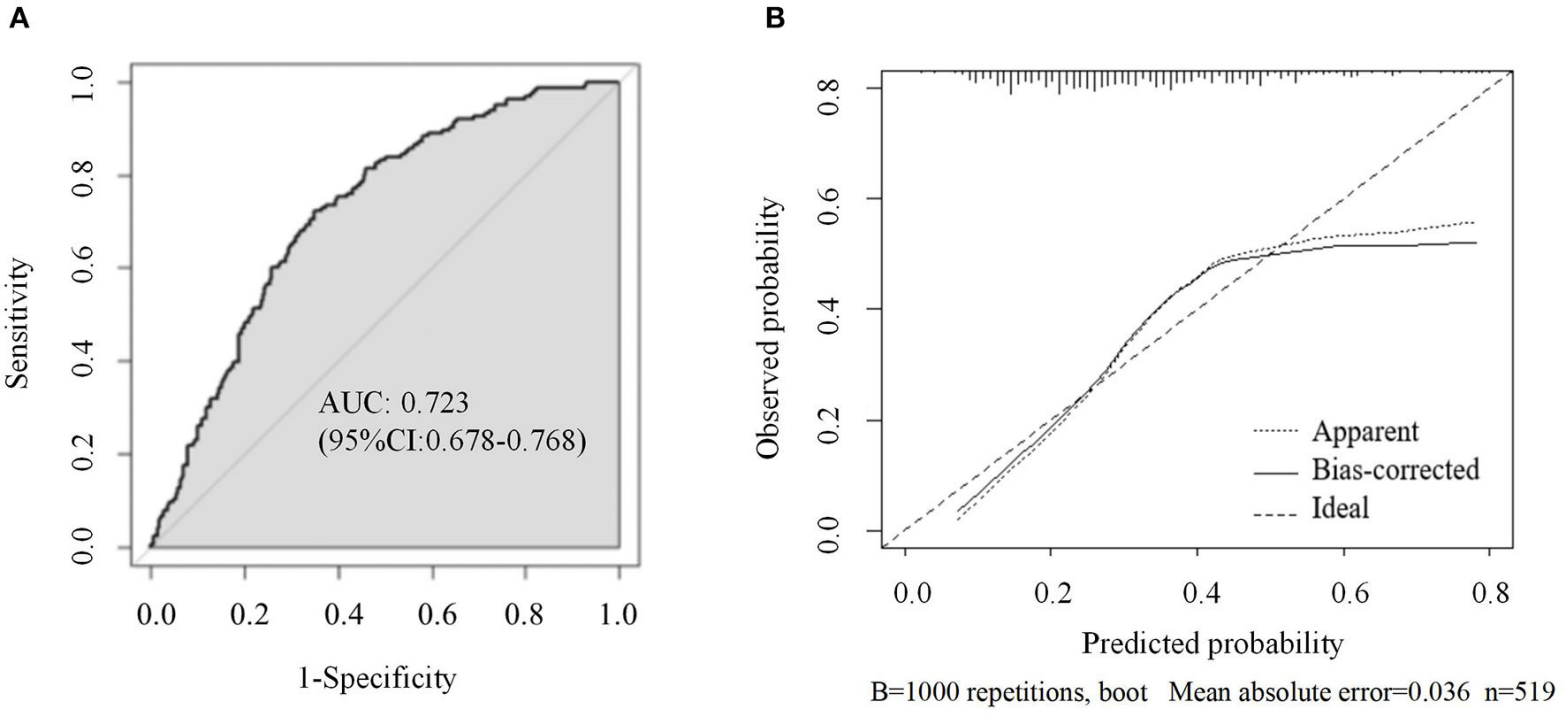
**(A)** ROC curve of the nomogram used for predicting PSD at 3 months after MAIS onset in Chinese patients; **(B)** Calibration curves for the nomogram used for predicting PSD at 3 months after MAIS onset. X-axis represents the nomogram-predicted probability of depression; Y-axis represents the actual depression probability. AUC, area under the curve; ROC, receiver operating characteristic.

Based on the results of the multivariable logistic regression analyses, a nomogram comprised of six important factors was constructed to predict the risk of PSD (Figure 1). Each of the six independent predictors was projected upward to the value of the “Points” on top to get a score ranging from 0 to 100. By adding up six scores, we can get a “Total Points,” which was positively correlated with the risk of PSD. In addition, C-index and a calibration curve were used for the validation of the nomogram. Equivalent to the area under curve (AUC) value, the C-index (0.723, 95% CI: 0.678–0.768) shows that the nomogram has a high discrimination ability. With a cut-off value of 0.308, the sensitivity and the specificity were 72.3 and 65.2%, respectively (Figure 2A). In addition, the calibration curve approximating the 45° diagonal was presented, which demonstrated the consistency between prediction and actual observation of the presence of PSD (Figure 2B).

## Discussion

In this multicenter prospective cohort study, 32% of patients were diagnosed with PSD at 3 months after MAIS onset, roughly the same prevalence as PSD after general stroke. Our study suggested that higher levels of IB, smoking, and physical activity were protective factors lowering the risk of PSD. Furthermore, lower MMSE scores, higher neuroticism scores, and longer hospitalization days were risk factors for PSD.

As a readily available routine biomarker, bilirubin is widely used to diagnose hepatobiliary diseases (22) and hemolytic diseases (23) in the clinical. There are three forms of bilirubin in the body: total bilirubin, direct bilirubin (conjugated bilirubin), and indirect bilirubin (unconjugated bilirubin). Indirect bilirubin, which is equal to total bilirubin minus direct bilirubin, accounts for about 96% of the bilirubin in normal plasma and is tightly bound to albumin to be transferred to the liver for conjugation (24).

Bilirubin, generated as the ultimate product of heme catabolism, has long been regarded as a potentially toxic substance to be of great harm to the human body (24, 25). Numerous previous studies have shown that the level of serum bilirubin positively correlated with stroke severity (26, 27) and poor prognoses (28) in acute ischemic stroke. Similar effects have also been observed in studies assessing the relationship between bilirubin and depression (29). Miyaoka et al. showed that high levels of biopyrrins (bilirubin oxidative metabolite) in the urine of psychiatric patients were correlated with depressive symptoms (29, 30). Tang et al. (25) showed that higher bilirubin levels may indicate higher levels of perceived stress, which can increase the risk of PSD.

Currently, however, increasing evidence suggests that bilirubin plays beneficial antioxidant, anti-inflammatory, and cytoprotective roles (31–33). These specific properties are vital for organs that do not have strong endogenous cytoprotective defenses, especially the human brain (24, 31). All types of bilirubin share the same antioxidant effect but only unbound, unconjugated bilirubin is effective in treatment during an ischemic stroke (24). This is because unconjugated bilirubin (indirect bilirubin) is lipid soluble and can cross the blood–brain barrier easily (34). A great deal of studies had found that acute ischemic stroke can lead to the generation of free radicals as well as strong oxidative stress, which is an important pathogenesis of PSD (30, 35, 36). As a kind of crucial central nervous system antioxidant, indirect bilirubin exerts powerful antioxidant protection against free radical damage in the acute stage of stroke (29, 31, 32). Stroke patients with lower baseline indirect bilirubin levels may be more vulnerable to oxidative damage in the brain and therefore more susceptible to developing a depressive mood, which is consistent with a previous study (37). In addition, the role of serum bilirubin levels in depression in non-stroke subjects has also been explored and low nocturnal bilirubin levels have been shown to be associated with winter seasonal depression (25, 38). Therefore, it is reasonable to conclude that serum indirect bilirubin level is closely related to PSD, and a potential possible mechanism is that indirect bilirubin protects the integrity of neurons by playing a role in anti-oxidative stress (31).

Previous studies have shown that physical activity can effectively prevent or reduce the incidence of depression (5, 39). In our study, physical activity was divided into two categories based on whether patients exercise moderately or vigorously every week, such as jogging. The endorphin hypothesis suggests that physical activity increases the secretion of endogenous opioids in the brain and the levels of β-endorphins, which act as analgesics and stimulants, thereby reducing depression levels. The monoamine hypothesis is another widely recognized hypothesis of the etiology of depression. It is believed that the occurrence of depression is caused by the decrease in the concentration or function of monoamine neurotransmitters in the synaptic clew of the central nervous system (40, 41). Moderate physical activity can not only promote brain microcirculation and improve the level of monoamine neurotransmitters in the brain but also can accelerate blood circulation and improve human metabolism, so as to achieve the purpose of controlling individual bad emotions.

It is generally known that smoking is harmful to people's health and increases the chance of getting lung cancer (42, 43). However, certain ingredients in cigarettes, such as nicotine, can stimulate the central nervous system and make people feel euphoric. This is because nicotine binds to nicotine-acetylcholine receptors in the brain, producing dopamine that gives smokers feelings of pleasure, excitement, and arousal (44). This can partly explain why smokers are less likely to develop PSD. Nevertheless, we do not advocate regulating anxiety and depression by smoking. We encourage preventing post-stroke depression through active exercise and healthy eating.

In addition, the length of hospital stay as an important predictor of PSD can reflect the severity of physical disability to some extent. In general, the longer the hospital stay, the higher the cost. Under the combined action of physical discomfort and great economic pressure, patients are very prone to depression.

Neuroticism is a personality trait characterized by negative emotions such as worry, guilt, loneliness, and vulnerability (45). Previous studies have confirmed that neuroticism is a risk factor for a variety of emotional problems, including depression (46, 47), but the underlying mechanism between neuroticism and depression remains unclear (48). In addition, studies have found that higher neuroticism is associated with poorer response to antidepressant treatment, longer treatment duration after depressive episodes, and a higher recurrence rate of depression (49). Therefore, personality testing can be used as a part of PSD screening, and more clinical attention should be given to stroke patients with neurotic personalities.

The MMSE is the best-known and commonly used short-screening tool to provide an overall measurement of cognitive impairment in clinical settings (50). Preliminary studies have shown that the MMSE scores of stroke patients with severe depression were significantly lower than those of nondepressed patients with similar background characteristics (51). From the perspective of anatomy, cognitive decline and depression may have the same location as brain lesions. This has been replicated in an independent study of stroke patients with left hemisphere lesions who were assessed during the first year after stroke (52). The relationship between cognitive decline and depression is strong while the specific mechanism still needs further research.

## Conclusion

A previous study found that the 3-month prevalence of PSD in the total stroke population was 39.7%. In this study, the 3-month prevalence of PSD in the mild stroke population was 32%. This suggested that the prevalence of PSD seems equally high even if the ischemic stroke is mild, which should be taken seriously by clinicians. In addition, our study found that a higher level of indirect bilirubin can lower the risk of PSD. This interesting finding may provide new insight into the importance of bilirubin, raising the possibility that modulating the level of serum bilirubin could be a potential novel approach for PSD treatment. Furthermore, compared with previous prediction models, our model is more comprehensive and novel by combining social demographic factors, subjective scale scores, and objective biological indicators. This nomogram may be conducive to the detection of patients with a high risk of PSD and early intervention to promote full recovery. In addition, further external validation through prospective, multicenter, large-scale trials of this model is also necessary.

## Data availability statement

The raw data supporting the conclusions of this article will be made available by the authors, without undue reservation.

## Ethics statement

The studies involving human participants were reviewed and approved by Tongji Medical College, Huazhong University of Science and Technology (Approved No. of Ethic Committee: TJ-IRB20171108). The patients/participants provided their written informed consent to participate in this study.

## Author contributions

ZZ led the study. YW performed the data analysis and implemented the methodology and prepared the original draft. WS, JM, WL, XQ, CP, GL, and YL collected the data. XZ and YX reviewed and edited the final manuscript. All authors contributed to the article and approved the submitted version.
